# An integrated model of environmental factors in adult asthma lung function and disease severity: a cross-sectional study

**DOI:** 10.1186/1476-069X-9-24

**Published:** 2010-05-20

**Authors:** Laura Trupin, John R Balmes, Hubert Chen, Mark D Eisner, S Katharine Hammond, Patricia P Katz, Fred Lurmann, Patricia J Quinlan, Peter S Thorne, Edward H Yelin, Paul D Blanc

**Affiliations:** 1 Department of Medicine, University of California San Francisco, San Francisco, CA USA; 2 Division of Environmental Health Sciences, School of Public Health, University of California Berkeley, Berkeley, CA USA; 3 Institute for Health Policy Studies, University of California San Francisco, San Francisco, CA USA; 4 Sonoma Technology Incorporated, Petaluma, CA USA; 5 Department of Occupational and Environmental Health, College of Public Health, University of Iowa, Iowa City, IA USA; 6 Cardiovascular Research Institute, University of California San Francisco, San Francisco, CA USA

## Abstract

**Background:**

Diverse environmental exposures, studied separately, have been linked to health outcomes in adult asthma, but integrated multi-factorial effects have not been modeled. We sought to evaluate the contribution of combined social and physical environmental exposures to adult asthma lung function and disease severity.

**Methods:**

Data on 176 subjects with asthma and/or rhinitis were collected via telephone interviews for sociodemographic factors and asthma severity (scored on a 0-28 point range). Dust, indoor air quality, antigen-specific IgE antibodies, and lung function (percent predicted FEV_1_) were assessed through home visits. Neighborhood socioeconomic status, proximity to traffic, land use, and ambient air quality data were linked to the individual-level data via residential geocoding. Multiple linear regression separately tested the explanatory power of five groups of environmental factors for the outcomes, percent predicted FEV_1 _and asthma severity. Final models retained all variables statistically associated (p < 0.20) with each of the two outcomes.

**Results:**

Mean FEV_1 _was 85.0 ± 18.6%; mean asthma severity score was 6.9 ± 5.6. Of 29 variables screened, 13 were retained in the final model of FEV_1 _(R^2 ^= 0.30; p < 0.001) and 15 for severity (R^2 ^= 0.16; p < 0.001), including factors from each of the five groups. Adding FEV_1 _as an independent variable to the severity model further increased its explanatory power (R^2 ^= 0.25).

**Conclusions:**

Multivariate models covering a range of individual and environmental factors explained nearly a third of FEV_1 _variability and, taking into account lung function, one quarter of variability in asthma severity. These data support an integrated approach to modeling adult asthma outcomes, including both the physical and the social environment.

## Background

The potential relationship of environmental factors to morbidity in asthma is highly complex and difficult to study. Environmental risk factors for developing asthma (such as ambient pollution, antigens, and indoor air quality) have been given considerable research attention, particularly in the onset of childhood disease. In contrast, the role of the environment in asthma severity and disease-related quality of life for those who have established asthma has been less well studied, particularly among adults.

As importantly, studies of environmental factors in relation to asthma morbidity frequently focus on only a subset of exposures. Certain studies of indoor environmental exposures have emphasized specific allergens, such as from house dust mites, pets, cockroach, and rodents [1-5] or inflammatory agents such as endotoxin or glucans [6]. Others have focused on other indoor factors, such as secondhand smoke [7,8], dampness [9], or combustion heating and cooking sources [10]. Outdoor physical environmental factors have also been studied in relation to asthma severity or morbidity, including ambient air pollution (both fine and coarse fraction particulate matter, NO_2_, and ozone), or, on the other hand, residential proximity to roadways and traffic density, although typically both subsets of exposures are not included in the same analysis [11-18]. Several comprehensive literature reviews have summarized the evidence for these environmental factors, but cannot address the interplay among the various kinds of exposures because of the limitations of the analyses noted above [19-21]. Beyond these topics, occupational factors in relation to asthma, such as workplace exposure to vapors, gas, dust, or fumes (VGDF), represent an entirely separate avenue of investigation [22-26]. Finally, social environmental factors, in particular socioeconomic status (SES) at both the individual and neighborhood level [27-30] have been implicated in asthma severity, but have been analyzed almost entirely as exposures distinct from physical environmental factors.

Thus, while the body of environmental research on this subject has demonstrated that asthma indeed can be affected by multiple factors, integrated approaches have not been used to simultaneously examine the combined effects of a range of physical and social environmental exposures acting together. The present analysis takes advantage of a well-characterized cohort of adults with asthma over a spectrum of disease severity for whom a broad range of physical and social environmental exposures have been assessed. We hypothesized that exposure-response modeling, taking into account an integrated and wide-ranging group of environmental factors, would demonstrate substantial explanatory power for lung function and asthma severity associated with these variables. Moreover, we anticipated that, combined together, these effects would be substantially greater than those manifested by any isolated subset of environmental factors. Underlying these hypotheses is a conceptual model in which heterogeneous environmental exposures can act individually, collectively in clustered groups and, potentially, interactively to affect asthma-related outcomes.

## Methods

### Overview

We incorporated data on a wide array of potential environmental risk factors for adverse outcomes collected in an ongoing cohort study of adults with asthma and/or chronic rhinitis. Data derive from three sources: structured telephone interviews, home visits with direct sampling of key measures, and secondary sources of data on air quality, land use, traffic, and the socioeconomic status of the immediate neighborhood linked via geocoding to each subject's residential address. The two principal outcomes were the percent predicted forced expiratory volume in one second (FEV_1_) and asthma severity, defined using a validated scoring metric that correlates with, but is independent of, lung function. In addition, asthma-specific quality of life was included as a secondary outcome.

In order to examine a wide range of potential exposures while minimizing the number of items tested, we prioritized for inclusion candidate risk factors based on previous analyses of this cohort. We also divided the exposures into categories that provided a hierarchical structure for an initial screening step executed prior to final model specification. These five categories are: sociodemographic and behavioral characteristics; household biologics, including selected antigens (and subject antibody status); indoor air quality; metal concentrations in house dust; and the ambient physical and surrounding socioeconomic environments. The study was approved by the University of California, San Francisco Committee on Human Research; all subjects provided informed consent prior to participation.

### Subject Recruitment and Follow-up

Subjects with asthma were recruited initially through a random sample of pulmonary and allergy specialty practices in northern California beginning in 1992, followed by an additional sample of family medicine specialty practices in 1996. Eligibility was based on clinical diagnostic criteria consistent with American Thoracic Society and American College of Chest Physician definitions. Patients with concomitant diagnoses of chronic bronchitis or emphysema were ineligible; there were no other exclusions based on disease factors such as duration, age of onset, or presence or absence of atopy. In 1999, a third group of subjects was recruited by random digit dialing in the community at large within the same northern California catchment area. For this sample, eligibility was based on respondent report of a physician's diagnosis of asthma, or (to ascertain rhinitis) a physician's diagnosis of allergic rhinitis, chronic sinusitis, hay fever, or chronic post nasal drip. Respondents reporting a diagnosis of COPD or emphysema were ineligible. Subjects in all three samples were between age 18 and 50 at enrollment. Interviews were conducted by telephone at enrollment and at 18 to 24 month intervals thereafter. Since the last recruitment, an average of 74% of eligible participants was re-interviewed in each wave of multiple follow-up interviews. Details about the enrollment and retention of cohort members have been reported previously [22,31-33].

### Sources of Data

The present analysis includes data from the telephone interviews and home visits conducted in 2002-2003. The interviews include information on asthma and rhinitis symptoms, medication use, measures of health status and asthma-specific quality of life, smoking status, sociodemographic characteristics, and employment. Details of the home visit have been previously reported [33]. In brief, a study nurse and a research assistant conducted a comprehensive survey of the home environment, performed spirometry testing on each subject, and collected additional medical and psychosocial data. Environmental data gathered during the home visit included measures of indoor air quality and dust samples from the bedroom and living room. A blood sample was collected for antibody testing to selected allergens.

Spirometry was performed with the EasyOne™ (ndd Medical Technologies, Chelmsford, MA) which meets ATS 1994 diagnostic standards for spirometry measurements [34]. Spirometry measurements, including FEV_1_, were obtained using a standard protocol conforming to ATS guidelines [35].

In addition to the primary data collection, the participants' residential addresses were linked to numerous external data sources through geocoding, in which latitude and longitude coordinates are assigned to each address using electronic street map databases. Geocoding was carried out by Sonoma Technology (Petaluma, CA, USA) using the TeleAtlas MultiNetTM USA (TAMN) roadway database (Tele Atlas, Lebanon, NH, USA), which contains detailed roadway and address information and high positional accuracy. TeleAtlas Eagle Geocoding Technology was used to locate addresses in the TAMN database, yielding a corresponding latitude and longitude coordinate pair. When necessary, addresses were verified using data sources, such as aerial photography from the U.S. Geological Survey and online address location services such as Yahoo!^® ^and MapQuest^®^. Analyses were performed using the Environmental Systems Research Institute (Redlands, CA) ArcGIS software. These methods as applied to this cohort have been previously reported [16,29].

The geocoded addresses were linked to the 2000 US Census for socioeconomic characteristics of the participants' neighborhoods; the California Air Resources Board Air Quality Data and the Environmental Protection Agency Air Quality Data Retrieval System for air pollution data; the Bay Area Air Quality Management District and the National Weather Service for climatic data; the Tele Atlas/Geographic Data Technology Dynamap database for traffic data; and the U.S. Geological Survey National Land Cover Dataset for land use data.

### Outcome Measures

The primary outcomes studied were percent predicted FEV_1 _based on equations from NHANES III that use separate predictive values for male and female non-Hispanic whites, Hispanics, and blacks [36], and the Severity of Asthma Score, a previously validated measure that incorporates current symptoms and medications as well as longer term indicators of severity, e.g., both hospitalization and intubation and past and chronic corticosteroid administration [31,37-39]. These latter items can contribute 16 out of 28 total possible points on the scale. Higher scores reflect greater asthma severity. The scale was administered to all subjects, including those reporting a physician's diagnosis of chronic rhinitis but not concomitant asthma. These rhinitic subjects generally score in the lowest range of the scale (e.g., ≤5 points) and are included in the cohort to represent very mild to subclinical asthma [32,33]. As a secondary outcome, we included the Marks Asthma Quality of Life score (AQOL), a validated asthma-specific 20-item instrument administered only to those with asthma, with a maximum score of 60 (higher scores indicate poorer AQOL) [40,41].

### Categories of Exposure

Table 1 includes a summary of the laboratory measures and contextual data included in this analysis. Details of all exposure measurements are described below.

**Table 1:** Summary of data sources for environmental exposures.

Data source and exposure	Measurement/analysis	Variable format
**Telephone interview**		
Secondhand smoke (SHS)	Validated self-report measure (48)	*Dichotomous: *<1 vs. ≥1 hour/day
Occupational exposure to vapors, gas, dusts, or fumes (VGDF)	Self-report checklist measure (47)	*Categoric: *compared to employed with no VGDF exposure (ref), and not employed
		
**Home visit**		
Wall dampness	Hand-held moisture meter	*Continuous: *percent moisture
House plants, air filter, wood heating source, dog in home	Check-list	*Dichotomous: *present vs. absent
**Laboratory measures, from samples collected at home visit**		
Total dust	Gravimetric weight	*Continuous: *bedroom sample
Can f1 antigen	ELISA	*Continuous: *concentration in μg/g dust (maximum of bedroom or living room sample)
Anti-Can f1 specific IgE	Immunocap	*Dichotomous: *elevated (≥25 kU/l) vs. not
Endotoxin	Limulus assay	*Dichotomous: *lowest quartile concentration in EU/mg dust (maximum of bedroom or living room sample) vs. all others
Glucan	Sandwich ELISA	*Dichotomous: *highest quartile concentration in μg/g dust (maximum of bedroom or living room sample) vs. all others
Metal concentrations (Cu, Fe, Mg, V, Zn)	Aqueous extracts by inductively coupled plasma-mass spectrometry	*Dichotomous: *highest quartile concentration in μg/g dust (bedroom sample) vs. all others
**Contextual data, matched to residential addresses via geocoding**		
Neighborhood socioeconomic characteristics	2000 Census, measured at the block group level	*Continuous: *2 factors derived from principal components analysis of 13 SES variables
Land use	U.S. Geological Survey National Land Cover Dataset	*Dichotomous: *Urban/built environment vs. agricultural/forested
Distance from residence to roadway	Tele Atlas/Geographic Data	*Continuous: *meters to nearest road
	Technology Dynamap database	*Continuous: *km to nearest major road
		
Air quality	Air pollution: CA Air Resources Board Air Quality Data, Environmental Protection Agency Air Quality Data Retrieval System	*Continuous*: 2 factors derived from principal components analysis of 8 variables: (ozone, PM_2.5_, PM_10-2.5_, NO_2_, summer and winter temperatures and wind speeds)
	Climate: Bay Area Air Quality Management District, National Weather Service	

Sociodemographic and Behavioral Characteristics. Information on these risk factors was derived from the telephone interviews. Sociodemographic variables examined included: age, sex, race (white, non-Hispanic vs. all others), education (post-secondary education vs. less educational achievement), annual household income ($40,000 or more vs. <$40,000), and employment status (not employed; currently employed with self-reported exposure to vapors, gas, dust, or fumes [VDGF] [42]; and currently employed without such exposure [the referent category]). Smoking was categorized as heavy (10 or more lifetime pack-years of exposure), light (fewer than 10 pack-years), or never smoked (the referent category). We did not examine current smoking status separately, because there were only eight (5%) current smokers in the group.

Household biologics and antigen-antibody status. These measures included: weight of dust sample from the bedroom, presence of dog in home, amount of dog antigen (Can f1) in the dust (see below), anti-Can f1 specific IgE > = 25 kU/l in the subject's serum (by ImmunoCAP assay; Unilab Corporation, San Jose, CA), and concentrations of glucan and endotoxin in the dust sample (see below). The selection of these variables for inclusion in this analysis was guided by the previously published analysis of the home environment data [33].

The bedroom dust sample was collected by a hand-held vacuum from the mattress pad and pillows, using a standard eight minute protocol. The samples were analyzed for Can f1 as well as cat (Fel d1), cockroach (Bla g1), and dust mite (Der d1 and p1) allergens by the laboratory of Dr. S. Katharine Hammond (University of California Berkeley), using ELISA. The dust samples were also analyzed for endotoxin and glucan by the laboratory of Dr. Peter S. Thorne (University of Iowa, Environmental Health Sciences Research Center). Endotoxin was analyzed using the kinetic chromogenic Limulus Amebocyte Lysate assay (Lonza, Inc., Walkersville, MD) as previously described [6]. Glucan was analyzed using a sandwich ELISA method developed at the University of Iowa.

Indoor Air Quality. These measures included presence of a household air filter or purifier, one or more houseplants, and the use of coal or wood for home heating. These factors were documented during a systematic survey carried out as part of the home visit. Wall dampness was quantified as the percent moisture, as measured by a NO-PINS Moisture Meter, Model CT100 (Electrophysics, London, Ontario, Canada). The selection of these variables from among 16 available IAQ measures was guided by our previously published analysis of the home environment [33]. We also included in this category subject report of hours of exposure to secondhand smoke (SHS), using a validated survey instrument [43]; this was dichotomized as <1 vs. ≥1 hour per day.

Concentrations of selected metals in house dust. Aqueous extracts of dust samples were analyzed for five elemental metals: copper, iron, magnesium, vanadium, and zinc, chosen because of their potential biological activity and also because they might serve as markers of other ambient or indoor exposures. To obtain the aqueous extracts, dust samples were weighed and placed in vortex tubes with 5 ml of PBS-T solution. After centrifuging, the supernatant was transferred to vials and frozen for later analysis. For analysis, these supernatants were digested with HNO_3 _(nitric acid) and H_2_O_2 _(hydrogen peroxide). They were then analyzed using inductively coupled plasma-mass spectrometry (ICP-MS; West Coast Analytical Services, Inc., Santa Fe Springs, CA). Acid extractions were available for a subset of the dust samples (n = 48); Spearman correlation coefficients for the two methods ranged from 0.23-0.55, depending on the metal quantified.

External environmental factors. These variables fell into two subgroups: (1) socioeconomic and social environmental characteristics of the neighborhood and (2) physical environmental factors. To assess the former, we linked subjects to corresponding 2000 U.S. Census data at the block-group level, which captures a fairly homogeneous residential area of 600 to 3,000 persons (see also general geocoding methods, above). The Census data included a series of variables selected a priori to represent area-level measures of socioeconomic status (SES): measures of income and poverty status; employment status; education; home value, age, and ownership; family configuration; and population density. Categorical variables were reported as the proportion with a given characteristic in the block group and continuous variables were reported as the median value across all observations, resulting in a distribution of values whose units comprised a set of proportions or medians. Because the set of variables were substantially inter-correlated, we carried out a principal components analysis as part of a previously published analysis [29]; the resulting two measures of neighborhood SES were used in the present analysis as well. The first census factor captures characteristics associated with low SES (e.g., lower income, education, occupational status, and home values); the second is consistent with 'suburban' characteristics (e.g., more recently constructed, owner-occupied homes in less densely populated census block groups).

Physical environmental factors included a series of air quality and climatic measures; distances to nearest roadway and nearest major roadway; and a general measure of land use (urban/built environment vs. agricultural or undeveloped area). There were eight ambient air pollutant and meteorological variables that we wished to study: average daily maximum ozone (O_3_) and nitrogen dioxide (NO_2_) levels; average 24-hour levels of both fine (<2.5 μm) and coarse (2.5-10 μm) particulate matter (PM); and average summer and winter temperatures and wind speeds. For the air pollutants and temperature data, monthly values were spatially interpolated from air quality monitoring stations to the subjects' residences (as geocoded), using inverse distance weighting from up to three monitoring stations in each interpolation. For pollutants, a maximum interpolation radius of 50 km was used; for temperature data, the maximum radius was 100 km; wind speed values were assigned using data from the closest monitoring station within 50 km. However, over 90% of all of these data points were gathered from monitoring stations within 25 km of participants' residences. Data linkages and interpolation were conducted by Sonoma Technology, as noted above. All monthly values were averaged over 2002-2003, corresponding with the data collection time period.

Because of the inter-relationships among the eight air quality and climate measures, we carried out a principal components analysis similar to the one described above for the area-level SES measures. The first two factors generated in this analysis explained approximately 66% of the total variability and were retained for the analysis. The first factor weighted positively and to a similar degree (0.44-0.50) on four variables: O_3_, PM_2.5_, coarse particulate (PM_10-2.5_) and summer temperature. No other weighting for this factor exceeded ± 0.25. Highest quartile observations for this factor in our data set mapped to the inland agricultural areas of northern California, specifically the Sacramento and upper San Joaquin valleys, reflecting higher levels of summer photochemical pollution in these areas. The second factor weighted positively and to a similar degree (0.45-0.49) on three variables: NO_2_, higher winter temperatures and summer wind speed, and negatively on winter wind speed (-0.39). No other weighting for this factor exceeded ± 0.29. Highest quartile observations for this factor mapped to the central and eastern portions of the San Francisco Bay Area and to some parts of the San Joaquin Valley, reflecting higher levels of winter stagnation pollution.

Following a methodology developed in a previously reported analysis, we used two distance-to-roadway metrics in the analyses: distance to nearest roadway of any type and distance to nearest major roadway, including interstates and state highways [16]. Straight line distances were calculated from the geocoded residential locations to nearest roadways of four types -- interstate highways (road class 1), state highways (road class 2), arterial roads (road class 3) and local roads (road class 4), with minimum distances set for each type, ranging from 30 m for class 1 roads to 10 m for class 3 or 4 roadways. The air quality measures assess exposure at a macro-environmental (area) level, in contrast to the measures of distance to roadway, which are specific to each residence in the sample. Studies from other cohorts have shown an independent traffic effect (measured by distance to roads) after adjustment for regional pollutant concentrations [44].

### Missing Data

Complete data were not available for all study subjects. Among the 390 subjects interviewed by telephone who still resided in northern California, 226 (58%) completed home visits. Twenty-five of these subjects did not have complete data from either the blood samples or dust collection analyses; an additional 25 subjects were missing data from one or more of the external data sources, leaving a final sample of 176 subjects (78% of those who completed a home visit). The 176 home visit participants with full data for this analysis did not differ significantly from the 50 home visit subjects with missing data on the basis of age, gender, education, income, severity of asthma score or percent predicted FEV_1 _(data not shown). Of the 176 subjects, AQOL scores were not obtained among 26 (15%) who reported rhinitis only without concomitant asthma.

### Statistical Analysis

We used a hierarchical approach in developing multiple linear regression models of percent predicted FEV_1 _and asthma severity, in order to reduce the number of explanatory variables in the models. This approach, along with the principal components analyses described above, addressed the limitations inherent in examining a large number of predictor variables that preclude the inclusion of all of them in a single model. We first examined the set of predictors in each category separately; any variable shown to be at least marginally associated (p < 0.20) with the outcome under consideration was retained for the final model for that outcome. If only one of a set of indicator variables (e.g., heavy but not light smoke exposure) met the criteria for inclusion, we included the full set in order to retain the correct variable structure in the final model. The final models for both percent predicted FEV_1 _and asthma severity score ultimately retained 16 covariates (out of a possible 32), although the sets of variables included were not identical in the two models. We inspected the final models for collinearity based on condition indices and variance inflation factors, as described in Belsey, Kuh, and Welsch [45]; collinearity did not appear to be a problem in either model. We then compared successive models in a hierarchical fashion, using nested F-tests to determine if the most recently added set of predictors added explanatory power to the existing model. In further analyses, we added percent predicted FEV_1 _as an explanatory variable in the severity model and we also tested the change in explanatory power from a model with FEV_1 _only to one with all the covariates. We also used the model of severity including FEV_1_to assess its explanatory power for the secondary outcome, AQOL. Lastly, we re-estimated the final models for both percent predicted FEV_1 _and asthma severity score, omitting subjects reporting only rhinitis from the sample. All analyses were conducted in SAS release 9.2 (Cary, NC).

## Results

### Subjects

The majority of the cohort was female, white non-Hispanic, well-educated, and middle to upper income: of 176 subjects, 127 (72%) were female, 29% were nonwhite, 14% had no post-secondary education, and 26% had household incomes under $40,000 per year (Table 2). More than three quarters of the sample were employed at the time of study and nearly half of these reported vapors, gas, dust, or fume (VGDF) exposure at their current job. Among the 57 subjects (32%) who reported ever smoking, most (23% of total) reported fewer than 10 pack-years of exposure. Table 2 also shows the distributions of all the indoor and outdoor environmental exposure measures used in the analysis. Cohort members lived primarily in urban or built environments (74%), as opposed to agricultural or forested areas. Their homes were an average of 15 ± 16 meters from the nearest road and 2.4 ± 3.2 kilometers from the nearest major road.

**Table 2:** Subject characteristics (n = 176) and environmental exposures.

Characteristics	n (%)	Mean ± sd (median, range)
Sociodemographic and behavioral		
Age (years)		45 ± 9 (47, 22-61)
Female	127 (72)	
High school education or less	24 (14)	
Nonwhite (vs. white nonHispanic)	51 (29)	
Household income <$40,000/year	45 (26)	
Currently working	133 (76)	
VGDF exposure on current job	64 (36)	
Ever smoker	57 (32)	
Light smoker (<10 pack years)	40 (23)	
Heavy smoker (≥10 pack years)	17 (10)	
		
Household biologics and antigen-antibody status		
Total dust weight from bedroom sample (g)		1.7 ± 2.2 (1, 0.0-12.6)
Dog owner	77 (44)	
Can f1 allergen (max BR/LR), μg/g dust		108 ± 268 (9, 0-2661)
Elevated anti-Can f1 specific IgE	28 (16)	
Endotoxin (mean BR/LR), EU/mg dust		68 ± 98 (42, 0-736)
Glucan (mean BR/LR), μg/g dust		0.3 ± 0.4 (0, 0.0-2.6)
		
Indoor air quality		
Wall dampness, % moisture		9 ± 3 (8, 5-21)
Any houseplants	146 (83)	
Air filter in home	46 (26)	
Wood heat (vs. electric or gas)	24 (14)	
Secondhand smoke exposure (SHS),		
≥1 hr. per day	44 (25)	
		
Metal concentrations in bedroom dust (μg/g dust)		
Copper		27 ± 37 (18, 3-367)
Iron		69 ± 128 (33, 0-1038)
Magnesium		864 ± 529 (745, 201-4167)
Vanadium		0.4 ± 0.8 (0, 0.0-8.1)
Zinc		84 ± 129 (46, 0-991)
		
External environment		
Census block group factors		
Factor 1: Low SES (working or unemployed)		0.1 ± 1.1 (0, -2.3 - 4.9)
Factor 2: Suburban homeowners		-0.1 ± 1.0 (0, -2.8 - 1.5)
Urban/built environment	130 (74)	
Distance (m) to nearest road		15 ± 16 (12, 0-121)
Distance (km) to nearest major road		2.4 ± 3.2 (1, 0.1-19)
Air quality factors		
Factor 1: Higher O_3_, PM_2.5_, PM_10-2.5 _& summer temperature		0.1 ± 1.7 (0, -2.6 - 4.3)
Factor 2: Higher NO_2_, summer wind speed & winter temperature; lower winter wind speed		0.0 ± 1.5 (0, -7.4 - 2.1)

Air Quality Factor 1 is concentrated in the northern Central Valley of California

Air Quality Factor 2 is concentrated in the eastern portion of the San Francisco Bay Area

VGDF = vapors, gas, dusts, or fumes; BR = Bedroom; LR = Livingroom-kitchen; SES = Socioeconomic status

Most subjects (71%) in the cohort reported a diagnosis of asthma concomitant with rhinitis, with approximately 15% reporting either condition alone (Table 3). Mean values of percent predicted FEV_1 _ranged from 98.9 ± 13.3% among rhinitic subjects to 79.4 ± 19.6% for those with asthma alone, with the combined asthma and rhinitis group intermediate in terms of lung function impairment, 83.4 ± 18.1%. The Severity of Asthma Score yielded a mean score of 8.2 ± 5.4 among those with both asthma and rhinitis; 1.1 ± 1.1 among those reporting rhinitis alone; and 6.7 ± 5.5 among those with asthma alone. Severity scores were moderately correlated with FEV_1 _in this sample (Pearson correlation coefficient, r = -0.44).

**Table 3:** Outcome measures by condition group.

Condition group	Sample size	Percent predicted FEV_1_	Severity of Asthma Score
		mean ± sd (range)	
Asthma alone	26	79.4 ± 19.6 (41-113)	6.7 ± 5.5 (0-23)
Asthma with rhinitis	125	83.4 ± 18.1 (24-129)	8.2 ± 5.4 (0-26)
Rhinitis alone	25	98.9 ± 13.3 (66-118)	1.1 ± 1.1 (0-3)
All subjects	176	85.0 ± 18.6 (24-129)	6.9 ± 5.6 (0-26)

Higher Severity of Asthma scores indicate greater severity.

### Individual Exposure-Response Models

The first multivariate models for percent predicted FEV_1 _and severity of asthma score included behavioral and sociodemographic characteristics of the subjects (Table 4). Older age was associated with both lower FEV_1 _and increased severity scores (p < 0.001 for each model). Having a high school education or less (vs. at least some college education) was marginally associated with lower FEV_1 _(p = 0.05), and employment with VGDF exposure (vs. employment without VGDF) was associated with increased severity (p = 0.03). This model explained 11% of the variability in percent predicted FEV_1 _and 5% of the variability in asthma severity scores. Using the a priori cut-point of p ≤ 0.20, we retained age, education, employment (two variables: currently employed with VGDF exposure; not employed), and smoking status (light and heavy) in the FEV_1 _model; age and employment status were retained in the asthma severity model.

**Table 4:** Asthma Outcomes: Multivariate Regression Models by Exposure Category.

Models and covariates	Percent predicted FEV_1_				Severity of Asthma Score		
	**β**	**se**	**p**		**β**	**se**	**p**
Model 1 (sociodemographic and behavioral)							
Age (years)	-0.79	0.16	<.001		0.17	0.05	<.001
Female	-2.85	3.00	0.34		0.02	0.93	0.99
High school education or less	-8.03	4.07	0.05		-1.00	1.26	0.43
Nonwhite (vs. white nonHispanic)	1.36	2.99	0.65		0.73	0.93	0.43
Family income <$40 K	-1.60	3.45	0.64		1.01	1.07	0.35
Not employed	-3.30	3.66	0.37		1.81	1.13	0.11
Currently employed, VGDF (ref: employed, no VGDF)	-3.98	3.09	0.20		2.10	0.96	0.03
Light smoker (<10 pack years)	2.89	3.36	0.39		-0.99	1.04	0.34
Heavy smoker (≥10 pack years) (ref: never smoked)	7.59	4.87	0.12		-0.55	1.51	0.71
Adj. R^2^	0.11				0.05		
							
Model 2 (household biologics, antigen-antibody status)							
Total dust wt bedroom (per 100 g)	-2.02	0.63	<.001		0.05	0.20	0.80
Dog owner	-1.92	3.13	0.54		1.42	0.97	0.15
Can f1 allergen (max BR/LR), μg/g dust	0.00	0.01	0.42		0.00	0.00	0.35
Elevated anti-Can f1 specific IgE	-11.47	3.67	<.001		3.05	1.14	0.01
Endotoxin, lowest quartile	3.15	3.26	0.34		0.01	1.01	0.99
Glucan, top quartile	-0.84	3.21	0.79		-1.27	1.00	0.21
Adj. R^2^	0.10				0.03 (null)		
							
Model 3 (indoor air quality)							
Wall dampness, % moisture	0.58	0.55	0.30		-0.24	0.16	0.14
Any houseplants	1.50	3.76	0.69		-2.04	1.09	0.06
Air filter in home	-4.14	3.20	0.20		2.49	0.93	0.01
Coal/wood heat (vs. electric or gas)	5.31	4.12	0.20		-2.46	1.19	0.04
ETS exposure (≥1 hr/day)	0.01	3.24	1.00		0.79	0.94	0.40
Adj. R^2^	<0.01 (null)				0.06		
							
Model 4 (metals in house dust, upper quartile)							
Copper	1.27	3.63	0.73		-1.81	1.08	0.09
Iron	2.23	3.38	0.51		-1.79	1.00	0.08
Magnesium	5.60	3.49	0.11		-0.78	1.04	0.46
Vanadium	1.86	3.25	0.57		0.37	0.97	0.70
Zinc	-0.73	3.72	0.84		1.94	1.11	0.08
Adj. R^2^	<0.01 (null)				0.02 (null)		
							
Model 5 (external environment)							
Census Block Group Factors							
Factor 1: Poor (working/unemployed)	-1.05	1.38	0.45		0.68	0.43	0.12
Factor 2: Suburban homeowners	-5.39	2.00	0.01		0.56	0.63	0.38
Distance (m) to nearest road	0.29	0.09	0.001		-0.05	0.03	0.09
Distance (km) to nearest major road	0.42	0.45	0.35		-0.13	0.14	0.37
Urban/built environment	5.93	3.68	0.11		-1.73	1.16	0.14
Air Quality Factors							
Factor 1: Higher ozone, PM_2.5_, PM_10-2.5 _& summer temperature	0.06	0.96	0.95		0.05	0.30	0.86
Factor 2: Higher NO_2_, summer wind speed & winter temperature; lower winter wind speed	-2.37	1.10	0.03		0.44	0.34	0.20
Adj. R^2^	0.11				0.03 (null)		

Higher Severity of Asthma scores indicate greater severity.

In the second model in Table 4, having an elevated level of anti-Can f1 specific IgE was associated with both a marked decrease in percent predicted FEV_1 _(p < 0.001) and an increased asthma severity score (p = 0.01). We also observed a decline of 2.2% in FEV_1 _for every 100 g increase in bedroom dust (p < 0.001). Overall, this second model explained 10% of the variability in percent predicted FEV_1_. In contrast, none of the other household biologics or antigen-antibody status variables tested was significant in the asthma severity model, which as a whole was no more predictive than chance (i.e., model p > 0.05). Although it did not meet the criterion for inclusion, we nonetheless retained dog ownership in models of both lung function and asthma severity score, given the role of anti-Can f1 specific IgE.

In contrast to the previous model, the indoor air quality variables (Table 4, Model 3) demonstrated greater explanatory power for asthma severity score than for percent predicted FEV_1_. Having an air filter in the home was associated with a higher asthma severity score (indicating greater severity, p = 0.01), while having wood heating was associated with a lower score. Taken together, the indoor air quality variables explained 6% of the variability in asthma severity score, but almost none of the variability in percent predicted FEV_1_. The variables for air filter and wood heating were retained in both models; the severity model also retained the variable "any houseplants."

The next model (Table 4, Model 4) included the concentrations of copper, iron, magnesium, vanadium, and zinc in the bedroom dust samples. Because of the skewed distributions of these variables, we dichotomized the concentrations at the top quartile. Neither the model for percent predicted FEV_1 _nor asthma severity score demonstrated good explanatory power overall (R^2 ^≤ 0.02; p > 0.05 for both). Nevertheless, consistent with the individual inclusion criterion of p < 0.20, magnesium was retained in the FEV_1 _model, while copper, iron, and zinc were retained for asthma severity.

The fifth model shown in Table 4 includes all the external environmental variables. These variables explained 11% of the variability in percent predicted FEV_1_. Census factor 2 (concentrated in suburban areas) and air quality factor 2 were both associated with lower FEV_1_; greater distance from the nearest roadway was associated with higher FEV_1_. Land use (urban/built environment vs. agricultural/forestry) was also retained in the final model. As a whole, the model performed poorly for asthma severity score (R^2 ^= 0.03; p > 0.05), although several variables met the inclusion criterion, including census factor 1 (reflecting lower SES), distance to nearest road, land use, and air quality factor 2 (reflecting winter stagnation pollution located regionally to the San Francisco Bay Area).

### Integrated Exposure-Response Models

The results for the final model of percent predicted FEV_1 _are shown in the first set of columns in Table 5. Numerous variables retained their associations with lung function in the final model, including age (i.e., a residual effect with age-adjusted FEV_1_), education, total dust weight of the collected sample, anti-Can f1 specific IgE (a marker of specific sensitization and of general atopic status), census factor 2 (suburban SES), distance to nearest roadway, and air quality factor 2 (a winter stagnation pollution pattern). Thus, sociodemographics, household biologics/atopic status, and the external environment all contributed approximately equally to the overall explanatory power of the model, with cumulative R^2 ^values initially 12%, increasing to 22%, and finally to 30%. Of note, the variables for indoor air quality and metal concentrations did not make significant contributions to the final model.

**Table 5:** Final Hierarchical Multivariate Models of Lung Function and Asthma Severity.

Models and covariates	Percent predicted FEV_1_				Severity of Asthma Score		
	**β**	**se**	**p**		**β**	**se**	**p**
**Sociodemographics and behavioral characteristics**							
Age (years)	**-0.56**	**0.15**	**<0.001**		**0.10**	**0.05**	**0.03**
High school education or less	**-9.25**	**3.67**	**0.01**				
Not employed	-4.48	3.14	0.16		1.75	1.05	0.10
Currently employed, VGDF (ref: employed, no VGDF)	-0.74	2.83	0.80		1.75	0.91	0.06
Light smoker (<10 pack years)	1.01	3.03	0.74				
Heavy smoker (≥10 pack years) (ref: never smoked)	3.76	4.40	0.39				
Adj. R^2 ^(cumulative)	0.12 *				0.07 *		
							
**Household biologics and antigen-antibody status**							
Total dust wt bedroom (per 100 g)	**-2.23**	**0.58**	**<0.001**				
Dog owner					0.80	0.85	0.34
Elevated anti-Can f1 specific IgE	**-9.79**	**3.28**	**0.003**		**2.81**	**1.08**	**0.01**
Adj. R^2 ^(cumulative)	0.22 *				0.10 *		
							
**Indoor air quality**							
Wall dampness, % moisture					-0.23	0.16	0.17
Any houseplants					-1.52	1.10	0.17
Air filter in home	-2.89	2.76	0.30		**1.95**	**0.89**	**0.03**
Coal/wood heat (vs. electric or gas)	1.31	3.82	0.73		-1.82	1.23	0.14
Adj. R^2 ^(cumulative)	0.21				0.14 *		
							
**Metals in BR dust (upper quartile)**							
Copper					-1.84	1.04	0.08
Iron					-1.67	0.95	0.08
Magnesium	4.40	2.85	0.13				
Zinc					1.26	1.05	0.23
Adj. R^2 ^(cumulative)	0.21				0.17 *		
							
**External environment**							
Census Block Group Factors							
Factor 1: Poor (working/unemployed)					0.36	0.39	0.35
Factor 2: Suburban homeowners	**-4.38**	**1.53**	**0.005**				
Distance (m) to nearest road	**0.28**	**0.08**	**<0.001**		-0.02	0.03	0.41
Urban/built environment	5.23	3.30	0.11		-1.44	1.03	0.16
Air Quality Factor 2: Higher NO_2_, summer wind speed, and winter temperature; lower winter wind speed	**-2.11**	**0.93**	**0.03**		0.28	0.30	0.36
Adj. R^2 ^(full model)	0.30 *				0.16		

* Added variables contribute significantly to explanatory power of model (p < 0.05) Parameter estimates and significance tests are from full model.

Cumulative R^2 ^calculated from models containing all preceding variables Higher Severity of Asthma scores indicate greater severity.

The parameter estimates for the final model of asthma severity score are shown in the second set of columns of Table 5. Older age, anti-Can f1 IgE, and having an air filter continued to be associated with increased severity. Moreover, each set of variables, with the exception of the final cluster of external environmental factors, contributed significantly to the explanatory power of the model. As a whole, the final model explained 16% of the total variability in the Severity of Asthma Score, approximately half of the model explanatory power for percent predicted FEV_1_. We re-estimated the final model of asthma severity, adding FEV_1 _(data not in Table). Lung function was significantly associated with severity score (p < 0.001) and added substantive additional explanatory power to the model, increasing the adjusted R^2 ^from 16% to 25%. Although FEV1 "absorbed" much of the variability in the covariates that had already been in the previous model, when these same covariates were added to a model of severity containing only FEV_1_, that new model also had significantly more explanatory power (adjusted R^2 ^increasing from 19% to 25%).

We also tested a secondary outcome, AQOL, among the 150 subjects with a diagnosis of asthma, using the final predictive model for SAS with FEV_1 _added as an additional independent variable. This model explained 22% of the variance in AQOL, with significant individual associations (p < 0.05) for percent predicted FEV_1 _(better lung function linked to improved status) and not working (linked to worse status). Lower area-level SES was marginally associated with poorer AQOL (p = 0.10). Of interest, however, two metals were statistically significant in this model and showed a marked association with AQOL scores: highest quartile iron (6.7 point score increment in the direction of worse status; p = 0.02) and highest quartile zinc (6.0 point score increment in the direction of worse status; p = 0.04).

As a sensitivity analysis, we re-estimated the final models for percent predicted FEV_1 _and asthma severity score after omitting subjects who reported rhinitis alone, without concomitant asthma. Although there was a slight attenuation in the effect sizes, both models were similar to the original models (data not shown).

## Discussion

In this analysis, we undertook a novel, global strategy to assess the environmental contribution to health status among adults with asthma and rhinitis. Although many investigators have studied the relationship of various specific environmental factors to asthma morbidity, most analyses focus on a single factor to the exclusion of others, for example, ambient air pollution, house dust antigens, road traffic exposure, or social environmental characteristics. Such approaches have yielded important observations on selected risk factors, but ignore by design the obvious reality that none of these exposures occurs in isolation.

By studying a wide ranging set of physical and social environmental factors taken together, we found that the sum was greater than the parts. The overall explanatory power of the models we tested was impressive: 30% for percent predicted FEV1 and 16% for severity of asthma score. When FEV_1 _was added to the latter model, indirectly incorporating the upstream environmental predictors of lung function as well, the explanatory power of the model increased to 25%. In terms of overall explanatory power, the model of AQOL was similar to that of disease severity.

Despite the global relationship to environmental factors, we observed no convincing consistent pattern of relationships across the different health outcomes for individual risk factors. Sociodemographics, household biologics, and the external physical and social environment all contributed to the variability in lung function. For asthma severity, demographics and the household measures were predominant, while the external environmental measures did not provide additional explanatory power. This heterogeneity, in and of itself, underscores the importance of an integrated approach to this complex problem since the picture appears different depending on the mix of predictors and the outcome studied. In contrast to the heterogeneity in results by health outcome, the results of the sensitivity analysis omitting patients with rhinitis alone were not substantively different from the main results, indicating that this subgroup did not bias the key findings.

With some notable exceptions, patterns we have seen in previous separate analyses of these exposures remained in the expanded models. Percent predicted FEV_1 _was associated with traffic-related exposures, with area-level SES, with quantity of house dust, and with specific IgE to dog allergen, but not dog ownership [16,29,33]. Asthma severity was associated with occupational VGDF exposure, with specific IgE for dog allergen and with having an air filter in the home (this most likely as a self-management response rather than as a causal factor contributing to severity). The fact that both outcomes are associated with specific IgE to dog, but not concentration of the allergen in dust or dog ownership most likely indicates that these findings are a marker for atopy, rather than representing a particular relationship with dog allergen. We have previously shown in this cohort that levels of IgE to other allergens (dust mite and cat) were moderately correlated with IgE to dog although the latter was associated most strongly with asthma outcomes (33). Consistent with prior analyses, asthma severity score was not associated with lower area-level SES after the inclusion of the individual sociodemographic measures.

Levels of endotoxin were similar to those reported in the U.S. National Survey of Lead and Allergens in Housing, where a strong relationship was observed between higher endotoxin and diagnosed asthma, asthma symptoms and asthma medication use [6]. However, in our study endotoxin was not associated with either asthma severity or lung function.

Some novel exposure measures included in this analysis did demonstrate associations with the primary or secondary outcomes. These include the combined air quality measures we developed through our principal components analysis, and metal concentrations obtained from bedroom dust samples. In the latter example, the highest quartiles of both zinc and iron, although marginal in models of the primary outcomes, were associated with significant decrements in asthma-specific quality of life. While it is possible that these associations are reflective of traffic-related pollution, there was no conclusive evidence for this in our study. This may point to an area of future investigations, as the relationships between metals and asthma outcomes have not been studied in household exposures.

The limitations of this approach are important to take into account. While the cohort has representation across the socioeconomic spectrum, there was a lack of concentration in poor urban areas where the prevalence of adult asthma can be higher. The lack of statistically significant effects of some exposures previously linked to asthma outcomes may be due to the relatively small sample size of this cohort. The size of the sample also precluded an investigation of interactive effects among the various categories of exposure. It is possible that the lack of complete data for 22% of the cohort could have introduced a bias, although there were no systematic differences between subjects with and without missing data. The localization of the study in northern California can be seen as a strength as well as a limitation. While the results may not be generalizable to other locales, the geographic concentration allowed for coherent interpretations of the air quality measures. The variable reduction techniques used for air pollution and neighborhood SES allowed us to examine multiple highly correlated variables in the same model, but negated the possibility of identifying the effect of any single exposure or characteristic. Given that actual exposures are almost invariably to multiple pollutants, not to single pollutants, a strength of our approach is that we can potentially capture more of the effects of the pollutant mixture and interactions with weather conditions than the usual single-pollutant or two-pollutant models. We also adopted strategies to avoid collinearity in other independent variables (for example, not including multiple specific serum IgE tests such as cat and dust mite in addition to dog, and reduction of continuous metals values to an ordinal categorization) and tested for collinearity among remaining covariates. Nonetheless, beyond the factor analyses that were included (area-level SES and air pollution exposure), we did not attempt further item reduction such as a formal assessment of clustering of risk factors, an approach that has been used in some studies [46]. Further, the cross-sectional nature of the study prevented us from distinguishing between pre-disposing, acute, or chronic effects of the exposures.

Another potential study limitation could arise from including predictor variables here that had already been identified in earlier analyses of this cohort; we have addressed this by combining environmental covariates that had not been studied together in the same model (for example, the indoor air variables, roadway distance, and area level SES). We also included entirely new factors in these analyses, such as ambient pollution and dust metal concentrations. Moreover, our strategy allowed for variable reduction at the outset, addressing multiple testing issues. The assessment of the contribution of each category of exposure was influenced by the hierarchy that we established a priori among the categories, moving from individual characteristics, to the indoor environment, and finally to the external environment. However, the similarity between the R^2 ^for the individual category models and the cumulative R^2 ^for the corresponding subsets of variables in the final model suggests that the general pattern would remain no matter how the categories were ordered. Finally, despite our best efforts to be as inclusive as possible, there may be other unmeasured environmental variables influencing lung function and asthma severity, including but not limited to potential gene-environment interactions.

## Conclusions

Multivariate models with variables from five different categories of potential individual and environmental risk factors explained 30% of lung function variability and, including lung function, 25% of variability in disease severity in adult asthma. This supports an integrated approach that takes into account both the physical and social environments. In practical terms, this can be applied in other field investigations of asthma by taking a more holistic approach to exposure assessment, including measures of biological, chemical, and societal factors at the individual, micro- and macro-environmental levels.

## Abbreviations

AQOL: Asthma Quality of Life; ELISA: enzyme-linked immunosorbent assay; FEV1: forced expiratory volume in one second; NO_2_: nitrogen dioxide; O_3_: ozone; PM: particulate matter; SES: socioeconomic status; SHS: secondhand smoke; VGDF: vapors, gas, dust, or fumes

## Competing interests

The authors declare that they have no competing interests.

## Authors' contributions

LT participated in the study design, conducted the analyses, and drafted the manuscript. JRB participated in the study design, guided the air quality analyses, and reviewed manuscript drafts. HC, MDE, PPK, PJQ, and EHY all contributed to the analysis and interpretation of the data and reviewed manuscript drafts. SKH conducted dust sample analyses for dog, cat, and dust mite allergens and reviewed manuscript drafts. FL provided all geocoded data from external data sources and conducted the data match. PST conducted dust sample analyses for glucan and endotoxin and reviewed manuscript drafts. PDB conceived of the study, participated in its design and coordination and helped to draft the manuscript. All authors read and approved the final manuscript.
